# Editorial: Metabolism and tumor microenvironment nexus in neuro-oncology

**DOI:** 10.3389/fimmu.2026.1832167

**Published:** 2026-04-01

**Authors:** Xian Wang, Siyi Wanggou, Uday Kishore

**Affiliations:** 1 Department of Mechanical and Materials Engineering, Queen’s University, Kingston, ON, Canada; 2 Ingenuity Labs Research Institute, Queen’s University, Kingston, ON, Canada; 3 Sinclair Cancer Research Institute, Queen’s University, Kingston, ON, Canada; 4 Department of Neurosurgery, Xiangya Hospital, Central South University, Changsha, Hunan, China; 5 Department of Veterinary Medicine (CAVM) and Zayed Centre for Health Sciences, United Arab Emirates University, Al-Ain, United Arab Emirates

**Keywords:** Combination therapeutics, glioblastoma, immunotherapy, neuro-oncology, tumor microenvironment

## Introduction

Various articles assembled in this Research Topic collectively reinforce a central concept in neuro-oncology: glioblastoma (GBM) and related central nervous system (CNS) malignancies are being understood as immunometabolic ecosystems. Across biophysical, mechanistic, translational, and bioinformatic investigations, a unifying theme emerges, that is, metabolic reprogramming, inflammatory signaling, and microenvironmental remodeling are inseparable processes that govern tumor initiation, immune response, and therapeutic resistance.

## A myeloid-enriched and metabolically conditioned microenvironment

The review by Lamorlette et al. synthesizes our current understanding of the GBM tumor microenvironment (TME) as a predominantly myeloid-enriched compartment. Tumor-associated macrophages (TAMs), bone marrow–derived monocytes, resident microglia, and myeloid-derived suppressor cells (MDSCs) together make a substantial fraction of tumor mass. These immune populations are described as a continuum of phenotypic states shaped by local cues, treatment history, and tumor-intrinsic factors. Although primarily focused on immunologic composition, this framework implicitly underscores metabolic constraints. TAMs support angiogenesis, glioma stem cell maintenance, and local immunosuppression, which metabolically adapts to hypoxia, nutrient competition, and oxidative stress. The continuum model of myeloid states aligns with the concept that immune cell phenotype in GBM is dynamically conditioned by the metabolic and structural features of the TME rather than fixed polarization programs.

## Chronic neuroinflammation as a pre-tumorigenic metabolic driver

Bao et al. extend the above-mentioned discussion by examining chronic neuroinflammation as a driver of gliomagenesis. The authors delineate how sustained activation of microglia and astrocytes remodels the CNS immune landscape, possibly via TLR signaling, NLRP3 inflammasome activation, IL-6/STAT3, and NF-κB pathways. Persistent inflammatory signaling promotes genomic instability, epigenetic dysregulation, and emergence of glioma-initiating cells. While the article focuses on immune signaling, these pathways are intrinsically linked to cellular metabolism. Chronic cytokine exposure alters mitochondrial function and increases reactive oxygen species. Blood–brain barrier disruption further permits peripheral immune infiltration, altering nutrient and cytokine gradients within the CNS. Thus, neuroinflammation shall be considered not solely an immune perturbation but also a sustained metabolic reprogramming event that precedes and facilitates malignant transformation.

## Pyroptosis and immune infiltration in reclassified GBM

Zheng et al. provide a bioinformatic and experimental perspective on pyroptosis-related gene signatures in WHO CNS5-defined GBM. By constructing a prognostic model incorporating NOD2 and PLCG1, the authors demonstrate that pyroptosis-associated signaling correlates with survival, immune infiltration patterns, and differential therapeutic response. Pyroptosis is an inflammatory form of programmed cell death mediated by inflammasome activation and gasdermin cleavage. The findings suggest that pyroptosis-related pathways are intertwined with the immune composition of GBM, including enhanced immune activity in higher-risk groups. However, increased immune infiltration does not necessarily lead to effective antitumor immunity; instead, it may reflect a pro-inflammatory yet immunosuppressive TME. Given that pyroptosis involves cytokine release and metabolic stress, its role in GBM likely intersects with both immune remodeling and metabolic perturbation within the TME.

## Immunomechanics: compression as an independent driver of TAM phenotype

Darantiere et al. study the effects of chronic compressive solid stress on macrophages. Using stress magnitudes comparable to those measured in GBM, the authors demonstrate that compression alone induces transcriptional, metabolic, and functional changes that resemble TAM phenotypes. Compressed macrophages exhibited altered gene expression, metabolic signatures as assessed by fluorescence imaging, and functional changes including increased phagocytic activity. Notably, the resulting phenotype did not conform strictly to canonical M1 or M2 features, reinforcing the concept of macrophage states as context-dependent and multi-dimensional. These data provide experimental evidence that mechanical forces within the GBM TME can directly shape immune metabolism and function. Elevated solid stress, hypoxia, and vascular collapse are well-characterized features of GBM. The study therefore suggests that immunosuppressive macrophage programming may arise not only from biochemical signaling but also from physical compression.

## Implications for immunotherapy and translational strategies

Focused on neuroendocrine neoplasms, a review of CAR-T therapy by Golounina et al. highlights translational barriers that limit applications of CAR-T therapy in solid tumors, including antigen heterogeneity, insufficient T-cell infiltration, immunosuppressive cytokine networks (e.g., TGF-β, IL-10), PD-L1–mediated checkpoint inhibition, and a hostile TME. These challenges are directly relevant to neuro-oncology, where checkpoint blockade and adoptive cell therapies have shown limited benefit in GBM. The emerging consensus is that durable treatment efficacy will require combinatorial and TME-directed strategies rather than antigen targeting alone.

## Concluding perspective

The contributions in this Research Topic converge on a systems-level insight: metabolism is not a downstream consequence of tumor progression but a central organizing principle of the neuro-oncologic microenvironment. Inflammation, immune infiltration, mechanical stress, and cell death programs are metabolically intertwined processes that collectively shape disease trajectory. Future progress in neuro-oncology will depend on integrating immune profiling, metabolic characterization, and biomechanical analysis into unified therapeutic frameworks. By viewing GBM and related CNS tumors as immunometabolic and immunomechanical ecosystems, we may identify new intervention points capable of transforming currently refractory malignancies into more treatable diseases.

